# Prevalence of K-RAS mutations and CA125 tumor marker in patients with ovarian carcinoma

**DOI:** 10.22088/cjim.11.1.75

**Published:** 2020

**Authors:** Nazanin Bagherlou, Safar Farajnia, Saber Zahri, Ali Dastranj Tabrizi, Atefeh Nazari

**Affiliations:** 1 Department of Biology, Faculty of Sciences, University of Mohaghegh Ardabili, Ardabil, Iran; 2 Women’s Reproduction Health Research Center, Tabriz University of Medical Science, Tabriz, Iran; 3 Drug Applied Research Center, Tabriz University of Medical Sciences, Tabriz, Iran; 4 Department of pathology, Tabriz University of Medical Science, Tabriz, Iran; 5 Biotechnology Research Center, Tabriz University of Medical Science, Tabriz, Iran

**Keywords:** CA125, Codon 12, k-ras, RFLP, Ovarian carcinoma

## Abstract

**Background:**

Ovarian carcinoma is one of the leading causes of cancer-related death among females. K-ras codon 12 mutations are commonly occurring mutations in different types of cancers and leads to resistance against anti-EGFR therapeutics. Hence, determination of mutations in k-ras gene is crucial for predicting response to anti-EGFR therapies. This study aimed to evaluate the prevalence of k-ras gene mutations and CA125 tumor marker in patients with ovarian carcinoma in Tabriz city.

**Methods:**

Genomic DNA was extracted from 67 tissues pertained to women with ovarian carcinoma. Mutations in codon 12 and 13 of k-ras gene were analyzed by Nested PCR and RFLP methods. Titer of CA125 tumor marker was determined by ELISA.

**Results:**

Among the 67 patients, 7 patients (10.4%) had mutation in k-ras codon 12 while none of them had mutation in k-ras codon 13. Based on the results, the frequency of various genotypes were 89.55%, 10.44%, and 0%, for GG, GA, and AA alleles, respectively. The* p-value* for stage I and Grade I tumors were 0.022 and 0.04, respectively, indicating a statistically significant correlation between codon 12 mutation and stage I and Grade I tumors. Furthermore, we found significant correlations among CA125 tumor marker titers and histological grade (p<0.000) and stage (p<0.000). The mean serum CA125 tumor marker levels in malignant carcinomas were 499.84 U/ml.

**Conclusion:**

The results in this study indicated high prevalence of k-ras codon 12 mutations and high titer of CA125 tumor marker in patients with ovarian carcinoma in the study region.

Around the world, ovarian carcinoma is one of the leading causes of cancer-related death among females. The gain of function mutations in proto-oncogenes and inactivating mutations in tumor suppressor genes caused by genetic abnormalities participate in the progression of various tumors. It has shown that RAS mutations can lead to a constitutive stimulation of autonomous growth and contribute to the carcinogenesis. In humans, different members of ras family have been found in particular types of malignant tumors; N-ras in low grade serous ovarian, melanoma and k-ras (v-ki-ras2 Kirsten sarcoma viral oncogene homolog) in colorectal carcinoma and non-small cell lung cancer (NSCLC) (1-4). Ras protein is located on the inner part of the plasma membrane of the cells and responsible for GTP hydrolysis. It is an important regulator of cell growth, transformation and apoptosis. Ras protein has a fundamental implication in various pathological processes including RAS, RAF, MEK, ERK pathway and RAS, PIK3CA, PTEN, AKT, and mTOR pathways. K-ras mutations are frequently seen in some of tissues such as colon, lung and pancreas; and recent studies have shown that genetic alterations of ras oncogenes play a crucial role in early carcinogenic events (5, 6) and the trigger of ovarian cancer. 

The ras proteins are small GTPases which play a critical role in downstream signaling pathways of EGFR. The GTPase cycles between their active state when binding to GTP and the inactive state when binding to GDP (7, 8). Loss of GTPase activity is caused by mutations in codon12 and codon 13 of the k-ras gene. It has shown that mutation in k-ras codon 12 leads to constitutively active Ras protein (5, 9). Conventional cancer treatment methods include the combination of radiation, surgery and chemotherapy that lead to relapses within 3 to 5 years after treatment. In recent years, target therapy using monoclonal antibodies and chemical tyrosine kinase inhibitors have shown high response rates in the treatment of EGFR overexpressing cancers. Tyrosine-specific protein kinases are classified into 2 families, receptor tyrosine kinase (RTK) and cytoplasmic proteins. RTKs contains an amino-terminal extracellular domain responsible for ligand binding. These transmembrane proteins have an important role in efficient phosphorylation of tyrosine amino acid residues in signal transduction pathways. They act primarily as receptor for different growth factors such as EGFR and PDGFR and their downstream signaling cascade contribute to the proliferation, apoptosis and differentiation of the cells (10). 

Anti-EGFR targeted therapy includes tyrosine kinase inhibitors (TKIs) and monoclonal antibodies that show high anti-tumor activity in some cancers by interrupting signaling pathway. Monoclonal antibodies such as Cetuximab, Trastuzumab, and Panitumumab block the signaling cascade by binding to the extracellular ectodomain of EGFR, preventing EGF ligand binding and the signaling cascade but tyrosine kinase inhibitors attach to the intracellular domain of EGFR and block the downstream effectors of EGFR signaling pathway. Patients with mutant k-ras gene in their tissues demonstrate poor outcome to anti-EGFR therapy, hence, it is a crucial prerequisite to determine the mutation status of patient’s ras gene before their subjection to anti-EGFR target therapy (3, 11-14). The aim of this study was to investigate the frequency of k-ras mutations as well as CA125 tumor marker level and their correlation to various histopathological characteristics such as stage and grades in ovarian carcinomas from northwest of Iran.

## Methods

**Patients and samples: **To investigate the role of k-ras gene in ovarian carcinoma, different subtypes of ovarian carcinoma were collected. Formalin-fixed paraffin-embedded specimens from 67 patients with ovarian carcinoma recovered from al-Zahra Hospital. All samples had confirmation of cancer based on histological features in hematoxylin-eosin-stained tissue sections. Clinical information including histopathological reports were obtained for all of the patients with ovarian carcinoma. In every 67 specimens, histopathological characteristics were determined including degree of differentiation (divided into 3 groups of poorly, moderately and well differentiated); pathological staging, familial cancer history and cell subtypes.

**DNA extraction from paraffin-embedded specimens: **Malignant tumor tissue enriched section with a minimum surface area of 8 or 10 μm thicknes was isolated from the paraffin-embedded specimen (3) and deparaffinized with xylene as described (15). To decrease the risk of molecular cross-contamination during sectioning, the microtome was cleaned after cutting each tissue sample. Briefly, deparaffinization was accomplished by washing the sectioned tissue with 1000 μL xylene three times and isolated by centrifugation for 10 minutes. Then the sections were washed with 1000 μL of ethanol 96% to remove residual xylene. DNA extraction was performed by resuspension of tissue section in 400 μL lysis buffer (10mM Tris, 1mM EDTA, 50mM NaCl) containing 2% SDS and 60 μg proteinase K. The lysates were incubated at 40℃ for 18 hour, then continued with phenol-chloroform extraction and ethanol precipitation. Finally, DNA precipitates were washed with 70% ethanol and dissolved in sterile distilled water after dying (15, 16).

**PCR genotyping: **Nested PCR was carried out to improve the sensitivity and specificity of detection. The DNA extracted from samples were first amplified using the primers for K-RAS codon12: Forward: 5’- GCCTGCTGAAAATGACTG-3’; and Reverse: 5’- CCAATCAAAATGCACAG AGAG -3’. Then, one microliter of PCR products was amplified using inner nested primers of F2 5’-ACTGAATAT AAACTTGTGGTAGTTGGACCT-3’ and R2: 5’-GGTACATTT CAGATAA CTTAACTTTC-3’. First PCR and inner nested PCR for k-ras codon13 was performed using the following primers; F1: 5’- GCCTGCTGAAAATGA CTG-3’ and R1: 5’- CCAAT CAAAATGCACAGAGAG -3’; F2: 5’- ATA TAA ACT TGT GGT AGT TCC AGCTGG-3’; R2: 5’- TCA AAG AAT GGT CCT GGA CC-3. PCR was performed in 25 μl reactions containing 2.5 μl of 10X PCR reaction buffer, 1.5mM Mgcl2_,_ 0.2 μM dNTPs, 0.4 μM primers, 100ng of template DNA and 1 unite of Taq DNA polymerase. 

For k-ras codon 12, 33 amplification cycles were performed under the following conditions; one cycle initial denaturation at 94℃ for 4 min followed by 15 cycle of 1 min denaturation at 94℃ , annealing 1min at 53℃, extension 30 sec at 72℃, with a final extension of 5 min at 72 ℃.. The program for second PCR consisted of 18 cycles denaturation for 1min at 94℃, annealing at 55℃ for 1min, extension at 72 ℃ for 30 sec with a final extension at 72℃ of 5 min. For k-ras codon 13, 34 amplification cycles were performed under the following conditions; one cycle initial denaturation at 94℃ for 4 min followed by 15 cycle of 1 min denaturation at 94℃ , annealing 45 sec at 53℃, extension 30 sec at 72℃, with a final extension of 5 min at 72 ℃. The program for second PCR consisted of 18 cycles denaturation for 1min at 94℃, annealing at 53℃ for 45 sec, extension at 72 ℃ for 30 sec with a final extension at 72℃ of 5 min. The primers were designed to introduce a BSTN1 restriction site for codon 12 and van 91 I restriction site for codon 13 wild type alleles (17). Forward primer integrated a C residue mismatch at the second site of codon 11. This created a BstNI restriction site in the amplicon after PCR amplification on normal allele but inactivated in PCR products from mutated samples. For codon 13, forward primer incorporated a C residue mismatch at the first and second position of codon 10 creating van 91 I restriction site in the wild-type allele.

For RFLP analysis, digestion was carried out with BstN1 and Van91I for codon 12 and codon 13, respectively (18, 19). The digestion products were analyzed by electrophoresis on 3 % agarose gel, in a tris-acetate-EDTA (TAE) buffer at 94 volts alongside with a 50 bp DNA ladder (Fermentas, Lithuania) (20, 21). RFLP analysis for k-ras codon12 led to the generation of two fragment of 225-bp and 25-bp after BSTN1 digestion on wild-type samples. Mutated k-ras codon 13 resulted in a 146-bp fragment, whereas wild-type genotype resulted in two strands of 119 bp and 27 bp. 

**Quantitative determination of the CA125 cancer antigen: **The serum samples from 40 patients with ovarian cancer were collected from the Al-Zahra Gynecology Hospital. The quantitation of CA 125 was carried out by human CA 125 ELISA kit (RayBiotech Company Inc. USA) according to the manufacturer’s recommendations (22). The minimum and maximum detectable dose of Human CA-125 were determined to be 0.6 U/ml until 1000 U/ml. one hundred µl of standard or samples were loaded onto the ELISA plate and incubated 2.5 hours at room temperature (RT). Unbound elements were washed out with wash solution, then 100 µl of biotinylated antibody were loaded on to each well, incubated 1 hour at RT. After washing, 100 microliter of streptavidin-conjugated HRP reagent were loaded to the wells, and the plate was incubated for 30 minutes at RT. After washing, color development was performed by HRP substrate incubation and the optical density (O.D.) at 450 nm was determined using a microplate reader (22). 

**Statistical analysis: **Chi-square test, correlation analysis and spearman test were carried out to demonstrate specific associations between k-ras mutation and CA125 levels with histopathological characteristics (23). The statistical analysis was accomplished by chi-square test using SPSS software and p<0.05 was considered statistically significant and in Spearman tests correlation coefficient between +0.6 to +1 indicates a perfect significant correlation.

## Results

**Figure 1:**
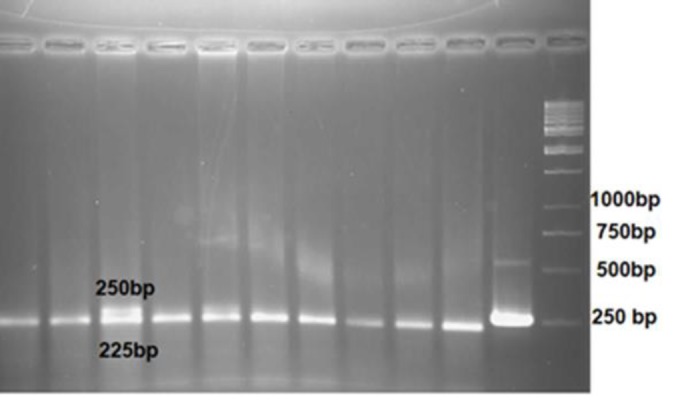
RFLP analysis for k-ras codon12 mutations

K-RAS codon12 and codon 13 mutation analysis were performed by PCR/RFLP technique in 67 patients with invasive ovarian epithelial carcinomas. The study was approved by the ethical committee of Tabriz University of Medical Sciences (IR.TBZMED.REC.1398.951). In our study, histopathological characteristics of the malignant tumor samples were collected for all patients, finally we detected the statistically significant correlation between codon12 k-ras mutations and the biological staging, cell differentiation and cell histopathological subtypes. The k-ras mutation gene fragment which carries a point mutation was distinguished from the wild-type k-ras gene fragment by the shifted mobility shown in the agarose gel in figure1.

**Analysis of codon 12 k-ras** **mutations in different types of** **ovarian carcinomas: **Results of PCR RFLP analysis revealed that 6 of 20 mucinous type malignant tumors and 1 of 47 serous ovarian carcinomas contained k-ras codon 12 mutation, respectively. These results indicate that k-ras codon 12 mutation are significantly frequent in mucinous type malignant tumors. 

Our study revealed that while 7 of 30 cases of stage I tumors contained k-ras codon 12 mutations, none of the samples belong to stage II, III, and IV had this mutation and while 6 of 25 cases of well differentiated tumors contained k-ras codon 12, one of the samples belong to moderately differentiated with this mutation.

The results indicated that k-ras mutation was significantly associated with lower grade, mucinous histological subtypes and lower stage of tumors. Compared to mucinous lesions, serous carcinoma exhibited low mutational frequency. Association of k-ras mutations with clinicopathological characteristic are shown in table 1 and genotype frequency for k-ras codon 12 mutation are summarized in table 2. 

**Analysis for mutation in codon 13 of the k-ras gene: **Results of PCR RFLP analysis revealed that none of 67 malignant tumors contained k-ras codon 13 mutation. These results indicate that k-ras codon 13 mutation are not significantly frequent in our ovarian cancer samples. The allelic frequency distribution of k-ras codon 12 and codon 13 mutations are summarized in table 3.

**Table 1:** Identification and distribution of codon 12 k-ras mutations

**Variables**	**GG** **(%)**	**GA** **(%)**	**AA** **(%)**	**Total**	**Pvalue**
Mucinous	14	6	0	20	0.001
Serous	46	1	0	47	
Stage I	23	7	0	30	0.022
Stage II, III, IV	37	0	0	37	
Well differentiated	19	6	0	25	0.04
Moderately differentiated	13	1	0	14	
Poorly differentiated	18	0	0	18	
Undifferentiated	10	0	0	10	

**Table 2:** Genotype frequency for k-ras codon 12 and codon13 mutation

	**PATIENTS=67**		
**Observed genotype frequency**	**Genotype**	**Genotype frequency**	**Observed genotype distribution**
Codon 12	GG	89.55	60
	GA	10.44	7
	AA	0	0
Codon 13	GG	100	67
	GA	0	0
	AA	0	0

**Table 3:** Allelic frequency distribution for k-ras codon 12 and codon13 mutation

	**N=134**	
**Observed allelic frequency**	**Allele**	**Number of alleles (observed frequency)**
Codon 12	G	127 (0.947)
	A	7(0.053)
Codon 13	G	134(1)
	A	0(0)

**Correlations between CA 125 levels and histolopathological characteristics; Grade, stage and subtype: **Sandwich ELISA method for determination of CA 125 tumor marker revealed that the mean serum CA125 tumor marker levels in malignant carcinomas were 499.84 U/ml (56 ranging from U/ml until 1500 U/ml), which were significantly higher than the normal titer of CA125 ranges. The serum CA125 tumor marker levels were also assessed for their correlation to the pathological stage, tumor grade, cell subtype and familial history (tables 4, 5, 6). We determined statistically significant correlations among CA 125 tumor marker titers and histological grade (p<0.000) and stage (p<0.000). 

No relationship was determined between CA 125 levels and tumor subtypes. Analysis for correlation with familial history revealed that CA 125 tumor marker shows a significant correlation with familial cancer history, but the correlation specifically with ovarian carcinoma was not significant (p=0.846).

**Table 4:** Correlations between CA 125 levels and histological grade, stage, subtypes and FM cancer history

CA125		GRADE	STAGE	SUBTYPE	FM Cancer history
Correlation COEFFICIENT SIG N	1 - 39	.648 .000 39	.756 .000 39	-.086 .604 39	-.035 .846 33
GRADE: Correlation COEFFICIENT SIG N	.684 .000 39	1 - 39	.601 .000 39	.010 .954 39	-.035 .846 33
STAGE: Correlation COEFFICIENT SIG N	.756 .000 39	.601 .000 39	1 - 39	.044 .792 39	.031 .863 33

**Table 5:** Mean of CA125 tumor marker levels

**Grade and stage factor**	**Number**	**Mean of CA125 levels**
Well differentiated; Grade I	11	**62.90 U/ml**
Stage I	14	**129.85 U/ml**
Moderately differentiated; Grade II	13	**577.76 U/ml**
StageII	11	**460.90 U/ml**
Poorly differentiated; Grade III	8	**876.62 U/ml**
Stage III	11	**1001.18 U/ml**
Un differentiated, Grade IV	7	**648.28 U/ml**
Stage IV	3	**524.33 U/ml**

**Table 6:** Spearman analysis between CA125 and histopathological characteristics

			**CA125**
Spearman's rho	CA125	Correlation Coefficient	1
		Sig. (2-tailed)	.000
		N	39
	Grade	Correlation Coefficient	.684^**^
		Sig. (2-tailed)	.000
		N	39
	Stage	Correlation Coefficient Sig. (2-tailed) N	.756^**^ .000 39

## Discussion

Epithelial ovarian carcinoma is a highly heterogeneous cancer with divergent clinical characteristics. This heterogeneity is not only reflected in the occurrence of different histological subtypes, but also appears in the carcinogenesis pathways (24, 25). EGFR-targeted therapy drugs like Cetuximab and Trastuzumab, which are already in the use for treatment of NSCLC and metastatic CRC, lead to a better prognosis and have recently been suggested as potential treatments for ovarian cancer because of the overexpression of EGFR in ovarian cancer (26). However, not all ovarian cancer patients have equally respond to these targeted treatments (24, 27). Considering the key role of k-ras mutation in EGFR signaling pathway, detecting of k-ras mutations in ovarian carcinoma should stand in the center of attention again, owing to the role of k-ras mutation as a predictive biomarker in poor response to anti-EGFR monoclonal antibody therapies (3, 6, 14, 28, 29).

Each of the histologic subtypes of EOC (epithelial ovarian carcinoma) is dependent on a divergent cell signaling pathways alterations. EOC is divided into two types, some of the tumors that are called Type I derived from borderline tumors including low grade serous and mucinous tumors which have high frequency mutations in k-ras, CTNNB1, PTEN, PIK3CA, BRAF, ERBB2 (25), but some of the tumors that are called Type II, derived from intraepithelial pathological cells in the fallopian tube and are characterized by TP53 mutations. Malignant epithelial ovarian carcinomas are divided into various histopathological subtypes containing endometrioid, clear cells, serous and mucinous, among these subtypes, the serous and mucinous subtypes are the most common types (30).

In our study, k-ras mutations were found in 30% of mucinous malignant tumors and in 2.12% of serous tumors. The results demonstrate a frequency of k-ras mutation similar to previous reports (6, 31-33) indicating the fact that k-ras mutations are commonly detected in low-grade mucinous carcinomas. These findings suggest that these changes may represent an early genetic alteration involved in the carcinogenesis of ovarian epithelial cells. The association of the k-ras mutation with mucinous tumors suggests that it plays a role in maintaining the mucinous differentiation pathways of ovarian epithelial cell (34). In a more recent study, researchers have detected that k-ras mutation is highly frequent in mucinous ovarian carcinomas as these mutations also accumulate in mucinous carcinomas obtained from other organs (35, 36). The results of our study indicated that k-ras mutation is significantly associated with differentiation grade and staging. In line with the previous consideration, we found a strong correlation between k-ras mutations and well-differentiated carcinomas (2, 31, 34, 37).

K-ras mutations have a high frequency in cancer tissues with FIGO I-II clinical stages, significantly but not in FIGO III-IV stages, hence most of the carcinomas with k-ras point mutations were in early clinical stages may indicate that k-ras mutations commonly occur as an early event in the development of carcinogenesis (2, 38). The circulating CA125 tumor marker is an antigenic determinant encoded by the MUC16 gene, which is expressed by epithelial ovarian tumors and various other pathological forms (39, 40). It is diagnosed by the monoclonal antibody OC125, which reacts with glycosylation-dependent antigens (41). CA125 is the best ovarian cancer early detection marker to date and the results of our study showed an association between CA125 levels and histopathological characteristics of tumors. These findings were consistent with several previous studies (42, 43). Jiang *et al.* described that serum CA 125 levels are relevant prognostic factors in cases of ovarian carcinoma (22) and reports that postoperative serum CA 125 levels are correlated with staging, cell differentiation and survival rate in cases of ovarian carcinoma (43).

In conclusion the results of this study indicated that K-ras mutation is a common event in ovarian carcinoma and more frequently present in carcinoma of mucinous histotype and lower FIGO stage. K-ras mutations could emerge as a pivotal factor for individually tailored anti-EGFR therapies. In addition, our conclusive results demonstrate correlation between Ca125 tumor marker titers and some of histopathological characteristics of ovarian carcinoma. The measuring of the CA125 tumor marker concentration in the serum of patients with ovarian cancer might be helpful for determining the grading and staging of the tumors and could be exploited for managing pharmaceutical treatment procedures. 

## References

[1] Fernandez-Medarde A, Santos E (2011). Ras in cancer and developmental diseases. Genes Cancer.

[2] Sadlecki P, Grzanka D, Grabiec M (2018). Testing for NRAS mutations in serous borderline ovarian tumors and low-grade serous ovarian carcinomas. Dis Markers.

[3] Guo F, Gong H, Zhao H (2018). Mutation status and prognostic values of KRAS, NRAS, BRAF and PIK3CA in 353 Chinese colorectal cancer patients. Sci Rep.

[4] Adderley H, Blackhall FH, Lindsay CR (2019). KRAS-mutant non-small cell lung cancer: Converging small molecules and immune checkpoint inhibition. EBio Med.

[5] Porru M, Pompili L, Caruso C (2018). Targeting KRAS in metastatic colorectal cancer: current strategies and emerging opportunities. J Exp Clin Cancer Res.

[6] Lee YJ, Lee MY, Ruan A (2016). Multipoint Kras oncogene mutations potentially indicate mucinous carcinoma on the entire spectrum of mucinous ovarian neoplasms. Oncotarget.

[7] Chen X, Yao H, Wang H (2019). Extending the Lifetime of Native GTP-Bound Ras for Site-Resolved NMR Measurements: Quantifying the Allosteric Dynamics. Angew Chem Int Ed Engl.

[8] Kano Y, Gebregiworgis T, Marshall CB (2019). Tyrosyl phosphorylation of KRAS stalls GTPase cycle via alteration of switch I and II conformation. Nat Commun.

[9] Hobbs GA, Der CJ, Rossman KL (2016). RAS isoforms and mutations in cancer at a glance. J Cell Sci.

[10] Lemmon MA, Schlessinger J (2010). Cell signaling by receptor tyrosine kinases. Cell.

[11] Auner V, Kriegshauser G, Tong D (2009). KRAS mutation analysis in ovarian samples using a high sensitivity biochip assay. BMC Cancer.

[12] Torres-Collado AX, Knott J, Jazirehi AR (2018). Reversal of resistance in targeted therapy of metastatic melanoma: lessons learned from vemurafenib (BRAF(V600E)-specific inhibitor). Cancers.

[13] Ferrer I, Zugazagoitia J, Herbertz S (2018). KRAS-Mutant non-small cell lung cancer: From biology to therapy. Lung Cancer (Amsterdam, Netherlands).

[14] Therkildsen C, Bergmann TK, Henrichsen-Schnack T, Ladelund S, Nilbert M (2014). The predictive value of KRAS, NRAS, BRAF, PIK3CA and PTEN for anti-EGFR treatment in metastatic colorectal cancer: A systematic review and meta-analysis. Acta Oncologica (Stockholm, Sweden).

[15] Yun BH, Xiao S, Yao L (2017). A rapid throughput method to extract DNA from formalin-fixed paraffin-embedded tissues for biomonitoring carcinogenic DNA adducts. Chem Res Toxicol.

[16] Yun BH, Yao L, Jelakovic B (2014). Formalin-fixed paraffin-embedded tissue as a source for quantitation of carcinogen DNA adducts: aristolochic acid as a prototype carcinogen. Carcinogenesis.

[17] Bornholdt J, Hansen J, Steiniche T (2008). K-ras mutations in sinonasal cancers in relation to wood dust exposure. BMC Cancer.

[18] Faleel FD, Zoysa MI, Lokuhetti MD (2016). Modified mismatch polymerase chain reaction-restriction fragment length polymorphism detected mutations in codon 12 and 13 of exon 2 of K-ras gene in colorectal cancer patients and its association with liver metastases: Data from a South Asian country. J Cancer Res Ther.

[19] Zhang H, Song J, Ren H (2013). Detection of low-abundance KRAS mutations in colorectal cancer using microfluidic capillary electrophoresis-based restriction fragment length polymorphism method with optimized assay conditions. PloS One.

[20] Diba K, Mirhendi H, Kordbacheh P (2014). Development of RFLP-PCR method for the identification of medically important Aspergillus species using single restriction enzyme MwoI. Braz J Microbiol.

[21] Mohammadi R, Abastabar M, Mirhendi H (2015). Use of restriction fragment length polymorphism to rapidly identify dermatophyte species related to dermatophytosis. Jundishapur J Microbiol.

[22] Jiang W, Huang R, Duan C (2013). Identification of five serum protein markers for detection of ovarian cancer by antibody arrays. PloS One.

[23] Cambruzzi E, Lima R, Teixeira S (2014). The relationship between serum levels of CA 125 and the degree of differentiation in ovarian neoplasms. J Bras Patol Med Lab.

[24] Ozer H, Yenicesu G, Arici S (2012). Immunohistochemistry with apoptotic-antiapoptotic proteins (p53, p21, bax, bcl-2), c-kit, telomerase, and metallothionein as a diagnostic aid in benign, borderline, and malignant serous and mucinous ovarian tumors. Diagn Pathol.

[25] Kurman RJ, Shih Ie M (2008). Pathogenesis of ovarian cancer: lessons from morphology and molecular biology and their clinical implications. Int J Gynecol Pathol.

[26] Palayekar MJ, Herzog TJ (2008). The emerging role of epidermal growth factor receptor inhibitors in ovarian cancer. Int J Gynecol Cancer.

[27] Smolle E, Taucher V, Pichler M (2013). Targeting signaling pathways in epithelial ovarian cancer. Int J Mol Sci.

[28] Shroff GS, de Groot PM, Papadimitrakopoulou VA, Truong MT, Carter BW (2018). Targeted therapy and immunotherapy in the treatment of non-small cell lung cancer. Radiol Clin North Am.

[29] Sideris M, Emin EI, Abdullah Z (2019). The role of KRAS in endometrial cancer: a mini-review. Anticancer Res.

[30] Alcázar JL, Utrilla-Layna J, Mínguez JÁ, Jurado M (2013). Clinical and ultrasound features of type I and type II epithelial ovarian cancer. Int J Gynecol Cancer.

[31] Hunter SM, Gorringe KL, Christie M (2012). Pre-invasive ovarian mucinous tumors are characterized by CDKN2A and RAS pathway aberrations. Clin Cancer Res.

[32] Anglesio MS, Kommoss S, Tolcher MC (2013). Molecular characterization of mucinous ovarian tumours supports a stratified treatment approach with HER2 targeting in 19% of carcinomas. J Pathol.

[33] Ricci F, Affatato R, Carrassa L (2018). Recent insights into mucinous ovarian carcinoma. Int J Mol Sci.

[34] Nodin B, Zendehrokh N, Sundstrom M (2013). Clinicopathological correlates and prognostic significance of KRAS mutation status in a pooled prospective cohort of epithelial ovarian cancer. Diagn Pathol.

[35] Liddell C, Droy-Dupre L, Metairie S (2017). Mapping clinicopathological entities within colorectal mucinous adenocarcinomas: a hierarchical clustering approach. Mod Pathol.

[36] Luo C, Cen S, Ding G (2019). Mucinous colorectal adenocarcinoma: clinical pathology and treatment options. Cancer Commun.

[37] Jones RP, Sutton PA, Evans JP (2017). Specific mutations in KRAS codon 12 are associated with worse overall survival in patients with advanced and recurrent colorectal cancer. Br J Cancer.

[38] Dias Carvalho P, Guimaraes CF, Cardoso AP (2018). KRAS oncogenic signaling extends beyond cancer cells to orchestrate the microenvironment. Cancer Res.

[39] Bruney L, Conley KC, Moss NM, Liu Y, Stack MS (2014). Membrane-type I matrix metalloproteinase-dependent ectodomain shedding of mucin16/ CA-125 on ovarian cancer cells modulates adhesion and invasion of peritoneal mesothelium. Biol Chem.

[40] Fortner RT, Schock H, Le Cornet C (2018). Ovarian cancer early detection by circulating CA125 in the context of anti-CA125 autoantibody levels: results from the EPIC cohort. Int J Cancer.

[41] Chen X, Li X, Wang X (2019). MUC16 impacts tumor proliferation and migration through cytoplasmic translocation of P120-catenin in epithelial ovarian cancer cells: an original research. BMC Cancer.

[42] Sanchez Vega JF, Murillo Bacilio MDR, Vintimilla Condoy AS (2018). Predictive equation of metastasis in patients with malignant ovarian epithelial tumors with the Ca-125 marker. BMC Cancer.

[43] Das C, Mukhopadhyay M, Ghosh T (2014). Correlation of cytohistlogical expression and serum level of ca125 in ovarian neoplasm. J Clin Diag Res.

